# Global Genome Biodiversity Network: saving a blueprint of the Tree of Life – a botanical perspective

**DOI:** 10.1093/aob/mcw121

**Published:** 2016-06-20

**Authors:** O. Seberg, G. Droege, K. Barker, J. A. Coddington, V. Funk, M. Gostel, G. Petersen, P. P. Smith

**Affiliations:** ^1^Natural History Museum of Denmark, Faculty of Science, University of Copenhagen, Sølvgade 83, opg. S, DK-1307 Copenhagen, Denmark; ^2^Botanic Garden and Botanical Museum Berlin-Dahlem, Freie Universität Berlin, Königin-Luise-Str. 6–8, D-14195 Berlin, Germany; ^3^National Museum of Natural History, Smithsonian Institution, Washington, DC 20560, USA; ^4^Botanic Gardens Conservation International, 199 Kew Road, Richmond TW9 3BW, Surrey, UK.

**Keywords:** Arboretum, biodiversity repository, biodiversity genomics, biobanks, botanic gardens, DNA banking, genome-quality samples, Global Genome Initiative

## Abstract

**Background** Genomic research depends upon access to DNA or tissue collected and preserved according to high-quality standards. At present, the collections in most natural history museums do not sufficiently address these standards, making them often hard or impossible to use for whole-genome sequencing or transcriptomics. In response to these challenges, natural history museums, herbaria, botanical gardens and other stakeholders have started to build high-quality biodiversity biobanks. Unfortunately, information about these collections remains fragmented, scattered and largely inaccessible. Without a central registry or even an overview of relevant institutions, it is difficult and time-consuming to locate the needed samples.

**Scope** The Global Genome Biodiversity Network (GGBN) was created to fill this vacuum by establishing a one-stop access point for locating samples meeting quality standards for genome-scale applications, while complying with national and international legislations and conventions. Increased accessibility to genomic samples will further genomic research and development, conserve genetic resources, help train the next generation of genome researchers and raise the visibility of biodiversity collections. Additionally, the availability of a data-sharing platform will facilitate identification of gaps in the collections, thereby empowering targeted sampling efforts, increasing the breadth and depth of preservation of genetic diversity. The GGBN is rapidly growing and currently has 41 members. The GGBN covers all branches of the Tree of Life, except humans, but here the focus is on a pilot project with emphasis on ‘harvesting’ the Tree of Life for vascular plant taxa to enable genome-level studies.

**Conclusion** While current efforts are centred on getting the existing samples of all GGBN members online, a pilot project, GGI-Gardens, has been launched as proof of concept. Over the next 6 years GGI-Gardens aims to add to the GGBN high-quality genetic material from at least one species from each of the approx. 460 vascular plant families and one species from half of the approx. 15 000 vascular plant genera.

## INTRODUCTION

The molecular revolution has changed almost every field of modern biology. Consequently the demand for access to biological samples appropriate for genomic research (quickly preserved, properly vouchered and correctly identified) by the scientific community has also increased significantly. This demand has been answered partially through growing efforts in fieldwork, in parallel with a supply of material from culture collections, seed banks, botanical gardens, zoos, aquaria and – to an increasing degree – material stored in natural history museums and herbaria. Improvements in sequencing technology have increased our ability to use traditional collections, but, despite the power of new molecular techniques, the use of traditional historical material frequently remains a challenge: (1) the DNA in specimens is often fragmented; (2) historical preservation techniques usually fail to inhibit endo- and exonuclease activity; or (3) the DNA has become almost inaccessible due to preservatives and fixatives that cause widespread post-mortem damage, interfering with sequencing (e.g. by cross-linking DNA and proteins in formalin-preserved tissues; see, Friedman and DeSalle, 2008; Zimmermann *et al.*, 2008). However, our ability to deal with these problems is quickly improving (see Der Sarkissian *et al.*, 2015), and, for certain applications, the degraded nature of DNA in museum samples is not an obstacle – as DNA does not have to be sheared prior to library building – and most, if not all, samples yield sequences suitable for either genome sequencing or plastomes and mitogenomes. As a consequence, a new field, museum genomics or museomics (Men *et al.*, 2008), has emerged, and the use of the collections has broadened beyond traditional uses in taxonomy and phylogenetics, to encompass new fields such as population genomics, adaptation genomics, phylogenomics, and ecological and conservation genomics.

Thus, to fulfil new research aims, and maximize the use of their collections with minimal destructive sampling, traditional biodiversity repositories have adapted increasingly to their new role and have started to store tissue or DNA samples to help overcome these barriers. Currently, storage is achieved in several different ways and, although storage in liquid nitrogen vapour might be considered the ‘gold’ or four-star standard (see Wong *et al.*, 2012; Spooner and Ruess, 2014), other storage methods such as tissue dried in silica or preserved in ethanol can be useful as well. However, the shear number of known and unknown eukaryotic species, 1·12–1·75 million and 8·7–12·25 million, respectively (lower estimates from Mora *et al.*, 2011; higher from Goombridge and Jenkins, 2002), significantly surpasses the capability of any single repository.

Though sample quality is important, it is not the only relevant parameter; sample diversity should also be considered. If we look closely, only truly charismatic vertebrate groups such as birds and mammals are ‘well’ represented in GenBank, i.e. there is at least one sequence from 8687 of all approx. 10 000 known bird species, and from 4587 of the approx. 5500 known mammal species (Chapman, 2009). This contrasts markedly with even one of the most charismatic seed plant groups, Orchidaceae, which is only represented by at least one sequence for 6265 species of the estimated approx. 22 500 species (Mabberly, 2008). The vertebrate groups have clearly received a disproportionate level of attention. The bias is even much stronger when it comes to more taxonomically diverse groups such as invertebrates, most vascular plants, green algae and fungi.

Consequently, the need for co-ordinated sampling efforts, storage and documentation strategies, as well as data and sample quality management is more urgent than ever. The only way to collect and proportionately sample the Tree of Life and salvage a genomic blueprint of key organisms for generations to come is to divide up this task globally and to make the resources available to the wider scientific community.

To face these challenges, the Global Genome Biodiversity Network (GGBN, www.ggi.si.edu/) was created in 2011 at an initial workshop in Washington, DC, hosted by the Global Genome Initiative (GGI, www.mnh.si.edu/ggi/). The goals of the two initiatives broadly overlap: the GGBN prioritizes professional care and data sharing of non-human genomic samples, including both legacy collections and phylogenetically informed sampling of biodiversity. The GGI’s mission is ‘to preserve and understand the genomic diversity of Life’, and aims at building a partnership of universities, research centres, government agencies, industry and museums from around the globe to achieve its mission. The GGBN thus provides the infrastructure required, *inter alia*, to achieve GGI’s mission. Of the 41 current GGBN members, 17 share their data via the GGBN Data Portal (data.ggbn.org; Droege *et al.*, 2014), though so far only a small fraction of their entire collections are databased. The GGBN is a member-driven organization based on a Memorandum of Co-operation, and is an unincorporated network of member organizations, which share the aim of making their high-quality, well-documented and vouchered biodiversity DNA and tissue samples discoverable for scientific research, by sharing a publically accessible, searchable database. Apart from building the infrastructure, the objective of the GGBN is to foster collaborations among biodiversity repositories in order to facilitate compliance with standards of quality, implement and improve best practices, secure interoperability and harmonize exchange of material in accordance with national and international legislation and conventions. Currently, the network focuses on DNA and tissue banks attached to traditional natural history or culture collections, but membership to the network is open to any biodiversity biobank (e.g. seed banks, environmental genebanks, zoos, aquaria and other types of biological repositories, as well as representatives of government, academic and other organizations involved in genomic biodiversity research). Members are expected to have interests in (1) genomic research and research infrastructure connected to biodiversity and the environment; (2) interacting with other members and the GGBN Secretariat; and (3) contributing to the achievement of the GGBN’s goals.

The GGBN’s primary goal is long-term storage and improving the discovery of tissue and DNA (genomic DNA), as well as the associated voucher specimens to allow verification of previous determinations in the future. For many taxonomic groups and applications, molecular based identification has become increasingly important with its potential for standardized, automated, scalable biodiversity identification. To reach that goal, well-documented reference databases are required to enable automated sequence comparison, e.g. by BLAST against nucleotide collection or primary sequence databases operating in the International Nucleotide Sequence Database Collaboration (INSDC; Cochrane *et al.*, 2011; see, however, Rach *et al.*, 2008). Presently, identification attempts often fail, primarily due to the lack of reference databases.

## THE GGBN PLATFORM

**Fig. 1.**
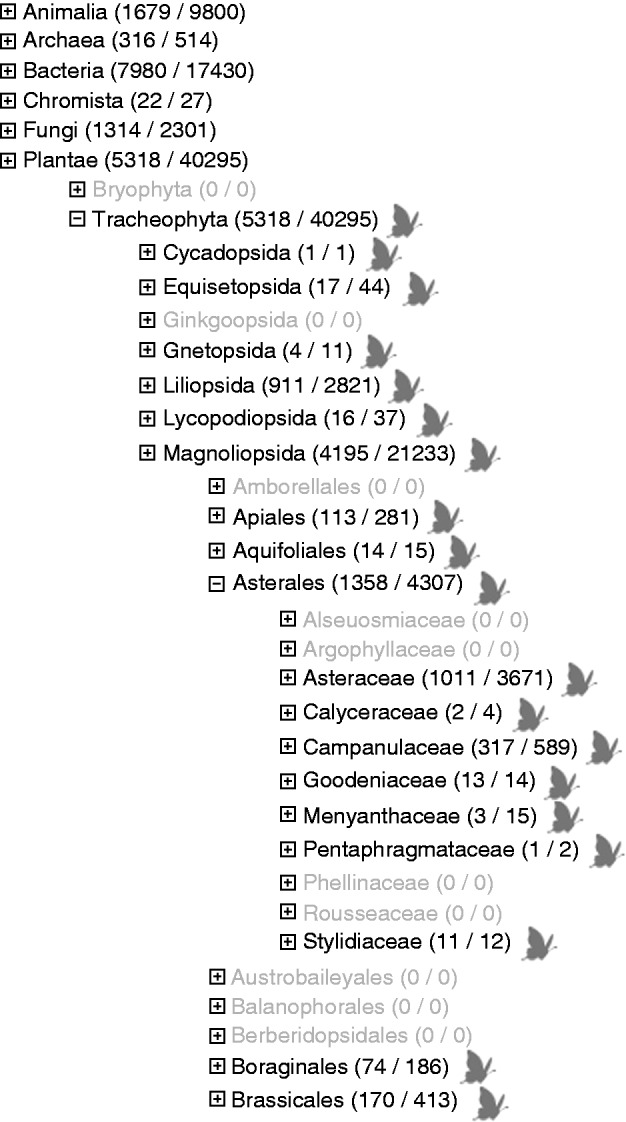
Tree view of GGBN records to facilitate gap analysis. The tree is based on combined taxonomic backbones (e.g. Catalogue of Life, GBIF backbone) and enables browsing through the Tree of Life to the species level. Visit data.ggbn.org/ggbn_portal/search/tree to get an overview of gaps at the GGBN. For example, Asteraceae (1011/3671) means that 1011 Asteraceae taxa and 3671 Asteraceae samples are available through the GGBN Data Portal. No Argophyllaceae are currently available (grey colour). The butterfly icon will point the user to the actual sample data.

The GGBN Data Portal (data.ggbn.org; Droege *et al.*, 2014) addresses and improves the use of samples and data by providing standardized access to genome-quality samples and related data from across the Tree of Life. The portal bridges the gap between biodiversity repositories, sequence databases and research by linking globally distributed biodiversity databases of genomic samples to vouchered specimens, sequence data and publications. This will: (1) allow a quick assessment of whether adequate samples are available and accessible for a specific project; (2) identify gaps in our current sampling of the Tree of Life (see Fig. 1); and (3) guide future strategic sampling, thus providing the necessary tools to save the genetic blueprint of threatened biodiversity. In addition, the Data Portal enables researchers worldwide to request DNA or tissue samples easily.

Best Practices and Standard Operating Procedures (SOPs) are required to document the processing of genomic samples correctly (e.g. sampling methods in the field), and different research communities and projects require specific materials, protocols and *a priori* knowledge. The planned GGBN Library (library.ggbn.org) will enable collaboration between different biodiversity sectors and will offer a platform to find and share relevant protocols and methods between communities.

**Fig. 2.**
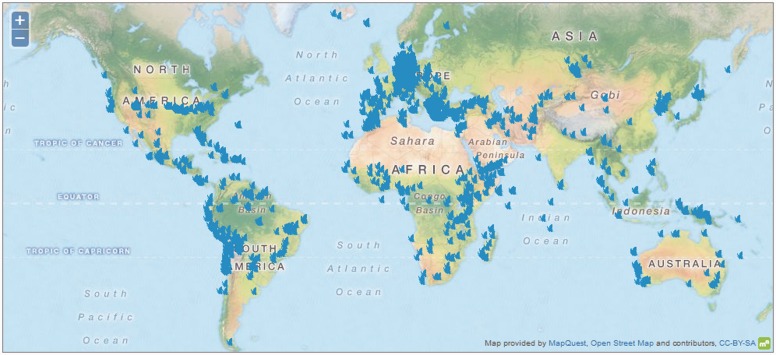
Geographic coverage of Plantae at the GGBN Data Portal in October 2015. Filling major taxonomic (see Fig. 1) and geographic gaps is one of the aims of the GGBN. This can only be achieved through collaborations within (e.g. GGI and barcoding initiatives).

The GGBN is still at the beginning of publicizing partner collections online (see Fig. 2), but important projects with several thousand samples are already available through the GGBN, e.g. Birds 10K Genome (Zhang *et al.*, 2014), birds within the Barcode of Life (Schindel *et al.*, 2011) and German Barcode of Life (GBOL; see Pietsch and Rulik, 2014), and the Genomic Encyclopedia of Bacteria and Archaea (GEBA; see Wu *et al.*, 2009).

### Legal and political considerations with respect to data sharing

The transfer of genetic sample information from initial collection and preservation in the field into collection management systems, along with voucher/strain and transaction/permit data, requires management. Adding and curating identifications made by taxonomic specialists, linking them to long-term sample storage systems and finally enhancing these with laboratory results is an additional responsibility.

The Nagoya Protocol came into force on 12 October 2014. The countries that have ratified it (cbd.int/abs/) are responsible for monitoring utilization by researchers and others under their jurisdiction, and for reporting through the ‘ABS Clearing-House’ (absch.cbd.int) on that utilization. Researchers, collection-holding institutions and networks ideally should adopt a common Best Practice approach to manage Access and Benefit Sharing (ABS). The GGBN has developed a Code of Conduct, recommendations for implementing Best Practices, and implementation tools, such as standard Material Transfer Agreements (MTAs) and mandatory and recommended data fields in collection databases. GGBN members are committed to adhere to Best Practice in relation to the Nagoya Protocol, and national laws and regulations relevant to ABS. The GGBN is collaborating with other organizations, such as the Consortium of European Taxonomic facilities (CETAF; www.cetaf.org), to harmonize Best Practices across overlapping scientific networks. Members are required to provide specific data and metadata fields in records to ensure that information regarding conditions of access and necessary legal documents are associated with the original specimen or sample records; this will both facilitate compliance with those conditions and enable efficient reporting on utilization. This will have implications beyond natural history collections and be of importance to the entire life science research community.

### A common data standard for a global network of biodiversity biobanks

Supporting documentation and enabling access to the rapidly growing collections distributed among the network members and facilitating communication about their content requires a globally agreed standard for sharing genomic data. The GGBN Data Standard (terms.tdwg.org/wiki/GGBN_Data_Standard) is a set of terms that has been developed based on existing community standards such as DarwinCore (Wieczorek *et al.*, 2012) and ABCD (Holetschek *et al.*, 2012) to represent preserved tissue and DNA information. It enhances accessibility of samples and their associated voucher specimen information for biodiversity research. The standard can be used by various approaches and is fully implemented in the GGBN Data Portal. Technical tools such as BioCASe (Holetschek *et al.*, 2009) and IPT (Robertson *et al.*, 2014) enable data providers to map their databases easily to the standard.

The GGBN considers it essential to bridge the gap to other communities to improve knowledge and data exchange or cross-references between different platforms such as the GGBN and the INSDC (e.g. the BioSample project, sequence submission automation). Consequently, the GGBN has submitted the GGBN Data Standard to the Genomic Standards Consortium (GSC) to be endorsed as an official GSC project and to the TDWG committee (Taxonomic Database Working Group; www.tdwg.org) to be ratified as an official data standard within the natural history collections community.

## WHAT CAN GGBN OFFER TO THE BOTANICAL COMMUNITY?

### Complementary strategies for *ex situ* conservation: seed banks vs. DNA banks

Vascular plants, particularly seed plants, are an important branch in the Tree of Life and an essential group from a human perspective, namely for economics, ecosystem function, health and nutrition. Seed plants not only provide us with the crops we eat and endless other commodities, but they are also an important component in most ecosystems. Conservation of seed plant diversity is the goal of many scientific communities and international conventions.

Seed banks have proven to be an effective and long-standing approach to preservation of plant genetic resources (Koo *et al.*, 2004). Preservation of plants important to human welfare, especially agriculture, is the core justification for most seed banks, and the history of seed banks spans centuries, preceding by a long time modern concerns with genome archives. Seed banks, first and foremost, intend to preserve viable samples. The Genetic Resource Information Network in the USA (GRIN; www.ars-grin.gov) maintains viable samples of > 2500 genera and 220 vascular plant families. The Millenium Seed Bank hosted by Kew Gardens (www.kew.org/science-conservation/collections/millennium-seed-bank) has samples of > 36 000 species. The Svalbard Global Seed Vault (www.regjeringen.no/en/topics/food-fisheries-and-agriculture/landbruk/svalbard-global-seed-vault) houses viable, duplicate samples of important crops from 4000 species to safeguard against accidental loss of diversity stored in traditional gene banks. These are but three examples of major seed bank initiatives across the globe. In contrast, all genomic samples stored in the GGBN are presumed dead and are ideally preserved using a means that halts all biological processes. Thus, these two storage strategies, viability vs. a genomic archive, encompassing plant diversity, complement and enrich each other as synergistic approaches to plant conservation.

### The role of botanic gardens

People have created botanical gardens all over the globe for >450 years; the oldest are in Pisa (1543), Padua (1545) and Firenze (1545) in northern Italy (www.bgci.org/resources/history). It is estimated that there are approx. 3000 botanical gardens world-wide, of which 1000 are members of Botanic Gardens Conservation International (BGCI; www.bgci.org). Together, these gardens collectively preserve an astonishing amount of living botanical diversity and play a considerable role in *ex situ* plant conservation (see Powledge, 2011 for a summary discussion). BGCI estimates that approx. 33 % of all approx. 353 000 known species are grown in gardens. Gardens are well-organized and inventoried institutions, and therefore co-operation and partnership to archive genome-quality samples of their holdings is an extraordinarily attractive and feasible proposition. While we have some idea of what taxa are available in gardens, greenhouses, arboreta and other living plant collections (hereafter referred to as ‘gardens’) via the BGCI website (www.bgci.org/plant_search.php), we do not have a complete overview of what is available in all of the thousands of gardens, spread across the globe. These gardens remain an obvious opportunity to increase our sampling coverage drastically. Thus a ‘gap analysis’ could be used to kick-start an effective, targeted, global sampling strategy with focus on what we do not have access to. These are precisely the goals of the Global Genome Initiative Gardens programme (GGI-Gardens), an initiative of several botanical institutions (see https://ggi.si.edu/ggi-gardens), which aims at sampling and preserving at least one living species from each of the approx. 460 vascular plant families and one species from half of the approx. 15 000 vascular plant genera (www.theplantlist.org) in 6 years. However, it is not the intention of GGI-Gardens to replicate the effort of existing repositories, such as crop gene and tissue banks, neither is it an aim to undertake population-level sampling of literally tens of thousands of vascular plant species. The goal is to build a collection of samples across the vascular plant Tree of Life that optimally samples the diversity at the generic level usable for genomic research.

### Data quality and sampling standards for living plant collections

**Fig. 3.**
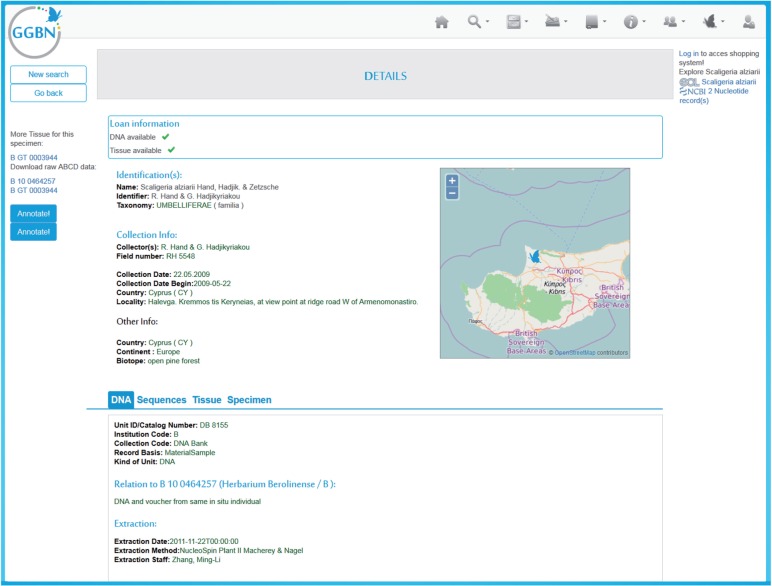
Record details for a plant DNA sample at the GGBN Data Portal. The shown DNA sample is an important reference for the description of *Scaligeria alziarii* Hand., Hadjik. & Zetzsche. The portal aggregates data from various sources to show collecting event details, associated tissues, voucher specimens and sequences (DNA tab highlighted). On the left, one finds information on other samples related to this specimen. External sources are queried live to get counts for this taxon. The blue on top contains information on loaning conditions of the DNA and tissue samples. (data.ggbn.org/ggbn_new/search/record?unitID=DB+8155&collectioncode=DNA+Bank&institutioncode=B).

All genome-quality samples should be vouchered (Funk *et al.*, 2005), preferably by a traditional herbarium specimen, and the vouchers should be deposited either in the individual garden’s own herbarium or in any recognized herbarium (i.e. registered in Index Herbariorum; sweetgum.nybg.org/science/ih/; Thiers, 2016). The genomic samples should be deposited in any botanical GGBN biodiversity repository, following an agreement with the involved institutions. Biorepository Best Practices recommend that, when possible, at least two samples of each species be collected from different sources and stored in two different herbaria and GGBN repositories (see Fig. 3). Beyond that, it is up to the individual gardens to duplicate species collections further.

Easy access to voucher metadata (e.g. name, location, co-ordinates) and the genomic sample are core goals of the GGBN, as are full compliance and transparency regarding all national and international conventions and legislation, including, but not restricted to, the Convention on Biological Diversity (www.cbd.int) including the Nagoya Protocol (www.cbd.int/abs) and the Convention on International Trade in Endangered Species of Wild Fauna and Flora (CITES; www.cbd.int). The metadata provide transparency regarding the original sources of the samples, thus making benefit sharing possible with the country of origin.

#### The GGI-Gardens Pilot Project

The potential of GGI-Gardens to cover plant diversity is impressive. In January 2015, GGI-Gardens established a collaboration to sample vascular plants from the mid-Atlantic region of the USA that included five partner gardens (Smithsonian Gardens and Department of Botany Greenhouse, US Botanic garden, US National Arboretum and the USDA Germplasm Farm). During a 10 week summer collection effort, two interns and a Smithsonian staff member were able to collect 158 families, 450 genera and 754 species. Sampling followed plant phenology – and standard herbarium practice – throughout the summer flowering season: flowering or fruiting individuals were priorities. Collections were vouchered (traditional herbarium specimens) and photographed; leaf material was preserved in both liquid nitrogen and silica gel. Generally, GGI aims at storing only a single sample of each species in liquid nitrogen plus 1–2 samples in silica gel. Storage in liquid nitrogen efficiently halts all biological life almost indefinitely and preserves all components in the cells, e.g. DNA, RNA and proteins, *in situ*. However, it is the most expensive form of storage and, given the number of species that GGI intends to collect, cost is a limiting factor. The vouchers from the pilot project are housed at the US herbarium, and liquid nitrogen- and silica-preserved leaf tissues are available in the Smithsonian’s Biorepository (http://data.ggbn.org/ggbn_portal/stats/details?registry=NMNH%2C+Washington). All data are available online through the GGBN (http://data.ggbn.org/ggbn_portal/search/result?kingdom=Plantae&institution=NMNH%2C+Washington), as is a draft of sampling protocol instructions (https://library.ggbn.org/share/s/GCFdZgavREaV03jySWJErw). Collecting has been done in the greenhouses throughout winter 2015 and spring 2016, and has led to a significant increase in the numbers collected and their diversity as the spring plants came into bloom (*Azalea*, *Cornus*, *Prunus*, etc.). Even given its initial ‘pilot’ scale, this is a cost-effective and high impact project. It can easily be scaled to international adoption by partner gardens. GGI-Gardens hosted a workshop at the 2016 GGBN meeting in Berlin (https://meetings.ggbn.org/conference/ggbn/2016/), where the programme was formally introduced and training offered to interested garden participants.

## CONCLUSION AND OUTLOOK

Since the beginning of the molecular revolution, obtaining access to DNA or tissue samples of sufficient quality has been a great challenge and rate-limiting step. However, the definition of ‘high quality’ is a constantly moving target. For lack of a co-ordinated global effort to archive truly ‘gold standard’ or four-star genomic samples, a lot of money, time and effort have been invested in extracting DNA from ever more degenerated samples. To store fully documented high-quality DNA and tissue samples for research to minimize these challenges, and thus enable the obvious and wide-ranging benefits of biodiversity genomic science are the main goals of the GGBN. One must acknowledge, however, that even though storage of fresh material in liquid nitrogen vapour is the current ‘gold standard’, other sampling and storage methods are also valuable, and may for a variety of pragmatic reasons be the only current option; live specimens and/or liquid nitrogen-stored material may be unavailable and the key taxa which are needed may exist only in remote, logistically challenging regions.

Presently, our knowledge of what samples are available, let alone our knowledge of their suitability for DNA extraction, is usually restricted to close-knit research groups or communities; information that is difficult for outsiders to access. iDigBio (Soltis and Godden, 2014) compiled a partial list of US biobank institutions (https://www.idigbio.org/genetic-resources), and the Global Registry of Biodiversity Repositories (GRBio; http://grbio.org/) compiled a similar global list, but neither database covers actual samples or searchable collections. Thus open information about what is currently stored in biodiversity biobanks is urgently needed for two reasons: (1) to further research and (2) to guide efforts and strategies in collecting and sampling new material. Through its Data Portal the GGBN makes biodiversity samples discoverable and accessible, thereby enabling ‘gap analysis’ and mobilization of the globally scattered DNA and tissue samples and their metadata. Access governed by standardized practices and policies compliant with the Convention on Biological Diversity will help make the intentions of the Nagoya Protocol a reality.

We envisage that the GGBN will grow rapidly into a self-sustaining entity, as institutions and scientists across the globe realize the importance of its ultimate goals − to share a blueprint of an intelligently sampled cross-section of the Tree of Life for current research and for the benefit of generations to come. Notable benefits to society will be realized through research and creation of a genomic knowledge infrastructure that contributes to human welfare, environmental monitoring and biodiversity conservation. We currently have the tools and best opportunities to do so – there is no excuse for not doing it.
